# Congruence between Hypothetical Willingness to Use Pre-Exposure Prophylaxis (PrEP) and Eligibility: An Online Survey among Belgian Men Having Sex with Men

**DOI:** 10.3390/ijerph16224411

**Published:** 2019-11-11

**Authors:** Johannes Bullinger, Thijs Reyniers, Bea Vuylsteke, Marie Laga, Christiana Nöstlinger

**Affiliations:** 1 Department of Public Health, Institute of Tropical Medicine, 2000 Antwerp, Belgium; johannesbullinger@yahoo.de (J.B.); treyniers@itg.be (T.R.); bvuylsteke@itg.be (B.V.); mlaga@itg.be (M.L.); 2 Faculty of Psychology, University of Vienna, Vienna 1010, Austria

**Keywords:** HIV-prevention, HIV, pre-exposure prophylaxis (PrEP), men who have sex with men (MSM)

## Abstract

Men who have sex with men (MSM) are at high risk for acquiring HIV in Belgium. This study explores MSMs’ hypothetical willingness to use pre-exposure prophylaxis (PrEP), assesses it against formal PrEP eligibility criteria, and identifies factors associated with incongruence between eligibility and willingness. We used data from an online survey of *n* = 1444 self-reported HIV-negative MSM. Participants were recruited through social media of MSM organizations and dating apps. Univariate analysis described PrEP willingness and eligibility; bivariate analyses examined how specific co-variates (socio-demographic, knowledge-related, and attitudinal and behavioral factors) were associated with eligibility and willingness. About 44% were eligible for PrEP and about 70% were willing to use it. Those who were eligible were significantly more likely be willing to take PrEP (*p* < 0.001). Two incongruent groups emerged: 16% of eligible participants were unwilling and 58% of ineligible participants were willing to use PrEP. Factors associated with this incongruence were sexual risk behavior, HIV risk perception, partner status, PrEP knowledge, and attitudinal factors. Because the two groups differ in terms of profiles, it is important to tailor HIV prevention and sexual health promotion to their needs. Among those at risk but not willing to take PrEP, misconceptions about PrEP, and adequate risk perception should be addressed.

## 1. Introduction

In Belgium and Western Europe, new HIV diagnoses have been declining for the last 10 years [1,2]. Men who have sex with men (MSM) are still at highest risk for HIV acquisition: In Belgium, more than half of new HIV infections were being diagnosed in this group in 2017 [1]. To further reduce the high number of HIV infections among MSM, primary prevention needs to be strengthened. 

Pre-exposure prophylaxis (PrEP), the use of antiretroviral treatment as prevention, has shown to be highly efficacious in reducing HIV infection risk, if used correctly [3,4]. Given this efficacy [4], PrEP-related research has increasingly focused on how to implement this novel biomedical prevention tool. In Europe, an increasing yet limited number of countries are now providing PrEP through national healthcare systems, including France, Norway, Belgium, Portugal, Luxembourg, Scotland, and Germany [5]. However, delivery and uptake may need to be upscaled and the implementation periods might have been too short to affect the overall course of the HIV epidemic across Europe [6]. 

Developing effective strategies for ensuring optimal PrEP uptake by populations at risk of HIV acquisition is an important implementation challenge. Ideally, such strategies should be based on active engagement of populations at risk, while also exploring PrEP use by individuals who are at lesser risk. Use of PrEP beyond the margins of clinical eligibility criteria may be costly [7,8,9], and may result in unnecessary exposures to potential side-effects. Another concern expressed on community-level has been prevention optimism, i.e., the belief that it is safe to engage in condomless sex because other men are perceived to take PrEP [10]. On the individual level, increased engagement in condomless anal sex, considered as ‘risk compensation’ may lead to more sexually transmitted infection (STIs) among MSM [11,12]. 

Willingness to take PrEP has frequently been used as a measure of acceptability and as a predictor of its uptake [13]. The construct “willingness” as part of a broader acceptability assessment has been investigated in other HIV-related health promotion areas, such as voluntary counseling and testing [14] and circumcision [15]. Examples outside the HIV field are prevention programs for cardiometabolic diseases [16] or mental health interventions [17]. Investigating this concept can contribute to better an understanding of how to disseminate theoretically promising public health interventions on a broader scale, translating efficacy into effectiveness. Willingness is believed to shape the pathway from behavioral intention to actual behavior, and it may partially predict actual behavior [18]. The perceived level of efficacy and barriers such as potential health consequences and social stigma are then typically presented as what may explain disparities between willingness and its actual uptake [18]. Studies conducted in high income countries showed PrEP acceptability rates among MSM between 40 and 60% [19]. Factors associated with willingness were younger age, having high HIV risk behavior (i.e., condomless anal intercourse (CAI) with casual sex partners, many partners) [20,21,22,23], and being aware of own HIV risk, for instance in Australia [23], England [24], and Germany [25]. Willingness was associated with previous post-exposure prophylaxis (PEP) use in Australia and England [23,24]. A study conducted in the United States found that men at highest risk (i.e., men of color, lower socio-economic status, and high HIV risk behavior) were most willing, but least likely to have access to PrEP [18]. MSM who reported to be unwilling to take PrEP, on the other hand, expressed concerns about side-effects, non-efficacy, lack of information, medical mistrust, and costs [26,27,28]. 

PrEP guidelines have issued eligibility criteria to identify individuals who qualify for PrEP based on known HIV risk factors [29,30], to ensure that PrEP is prescribed in a targeted way [31]. In Belgium, as in many Western countries, PrEP eligibility criteria for prescription and reimbursement issued by the Belgian Federal Office of Health include MSM with risky sexual behavior, people who inject drugs and share needles, sex workers, other people that may be exposed to greater HIV risk, and partners of HIV positive people whose viral load is detectable (see Box 1) [32]. A recent Belgian PrEP demonstration project showed that MSM at highest risk for HIV acquisition could be reached using similar screening criteria [33]. By November 2018, nine months after implementation of the Belgian prescription and reimbursement policy, 1352 PrEP users, predominantly MSM, were reported by the specialized HIV treatment centers qualified for PrEP delivery [34,35]. The willingness to take PrEP in the future so far has not been assessed against formal eligibility criteria. A better understanding of this relationship and its associated factors can inform tailored PrEP promotion and support strategies to optimize PrEP uptake.

Box 1 Pre-exposure prophylaxis (PrEP)-eligibility criteria in Belgium * (* Meeting only one criterion qualifies as being eligible. Source: Rijksinstituut voor Ziekte [32]).

**Criteria for men who have sex with men (MSM), that Permit Reimbursement of PrEP:**

(1) Condomless anal intercourse (CAI) with at least two different partners in the last six months.

(2) Diagnosed with multiple sexually transmitted diseases in the last year.

(3) Taken multiple PEP treatments in the last 12 months.

(4) Used psychoactive substances while involved in sexual activities.

**General PrEP eligibility criteria independently from sexuality:**

(1) People who inject drugs.

(2) Sex workers.

(3) Individuals that are being exposed to unprotected sex and a high risk of HIV.

(4) Partners of HIV-positive patients who has a detectable viral load.

The aim of this study was to explore hypothetical willingness to take PrEP among MSM, and to assess it against the formal PrEP eligibility criteria. More specifically, we aimed to assess differences in terms of socio-demographic, knowledge-related, attitudinal, and behavioral factors between MSM who are eligible and willing to use PrEP and those who display incongruences between their eligibility and willingness. 

## 2. Methods 

### 2.1. Study Design

A cross-sectional study was conducted among a convenience sample of Belgian MSM, using an on-line questionnaire. 

### 2.2. Study Population and Recruitment

The online questionnaire was promoted via social and sexual networking applications (e.g., Grindr or Hornet). Additionally, it was disseminated via social media of MSM community-based organizations in Belgium. It was online from 21st November 2016 to 27th February 2017, i.e., before PrEP was available for prescription and reimbursement. Inclusion criteria in this study were MSM or transgender; aged 16 years and above; self-reporting to be HIV negative or unknown serostatus; and living in Belgium or having a Belgian citizenship. 

### 2.3. Questionnaire and Variables Measured

We developed a questionnaire via SoSci [36]. The questionnaire was intentionally kept short and included skip logics and filter options for non-applicable questions to limit the time needed to complete it. Questions about socio-demographics, sexual behavior, HIV risk, and protective behaviors were similar to those used in other PrEP research, i.e., the Belgian PrEP demonstration project “Be-PrEP-ared” [33,37]. To inquire about PrEP awareness, knowledge, and acceptability, we adapted questions from similar research among healthcare providers [38,39]. It was available in Dutch, French, and English to reduce potential language barriers, and was piloted for feasibility and user-friendliness within the research team. 

We measured willingness to use PrEP using the following statement: “If PrEP was available in Belgium, what is the probability that you would use PrEP?”; answering options were given on a five-point Likert scale ranging from ‘certainly not’, ‘rather not’, ‘no opinion’, ‘rather yes’, to ‘certainly yes’. Answers ‘rather yes’ and ‘certainly yes’ indicated being willing to use PrEP. Any other answer denoted the absence of such willingness.

Eligibility criteria were measured with questions assessing the relevant sexual and preventive behaviors as defined by the Belgian criteria [32]. To calculate eligibility, we focused on criteria specific for MSM (see Box 1). The questionnaire included questions on preventive and sexual behavior, asking about HIV test recency, PEP and PrEP use, use of psychoactive drugs during sexual activity, number of anal sexual partners (with or without condom) in the last six months and anticipated CAI in the next three months. 

Sociodemographic items collected information on age, sexuality, nationality, place of residence, education, and relationship status. 

Five items measured participants’ attitudes towards PrEP through five-point Likert scales ranging from −2 ‘totally disagree’ to +2 ‘totally agree’. For the current analysis, the scales were dichotomized, where +2 and +1 denote ‘agree’, whereas −2, −1, and 0 denoted an absence of agreement with the respective attitudinal statements. Cronbach’s alpha for these five items was 0.68, hence we did not treat these items as one single scale.

PrEP awareness was examined through a dichotomous ‘yes’ or ‘no’ question, asking whether participants had ever heard of PrEP. Participants also had to self-estimate their knowledge about PrEP on a four-point scale from ‘very bad’ to ‘very good’. For the current analysis, the self-ratings ‘very bad’ and ‘rather bad’ were merged into the category ‘little knowledge’ and ‘rather good’ and ‘very good’ into ‘good knowledge’. 

Self-perceived risk to acquire HIV was also measured through a five-point Likert scale ranging from ‘very little risk’ to ‘very high risk’. Again, the middle category was added to the category implying absence of perceived risk.

### 2.4. Statistical Analysis

In this analysis, only completed questionnaires of participants matching the inclusion criteria for MSM were included. We analyzed cleaned data using IBM SPSS Versions 22.0 and 25.0 (IBM, Armonk, NY, USA). After forming four groups of participants according to their willingness and eligibility (group one: Eligible and unwilling to take PrEP; group two: Eligible and willing to take PrEP; group three: Ineligible and willing to take PrEP; and group four: Ineligible and unwilling to take PrEP), factors associated with either of the four groups were examined. We used a Chi-square test to examine the relationship between eligibility and willingness, and Chi-square or Fisher’s Exact Tests to determine the relationships between the four groups and potentially associated factors (preventive and sexual behavior, PrEP knowledge and attitudes). Statistical significance was set at *p* < 0.05.

### 2.5. Ethics

We obtained ethical approval for the study through the institutional review board of the Institute for Tropical Medicine Antwerp [1140/16]. Before filling in the questionnaire, participants were informed about the study, the procedures, and voluntary nature of study participation. By clicking through, participants consented to participate.

## 3. Results

### 3.1. Description of Study Sample 

**Figure 1:**
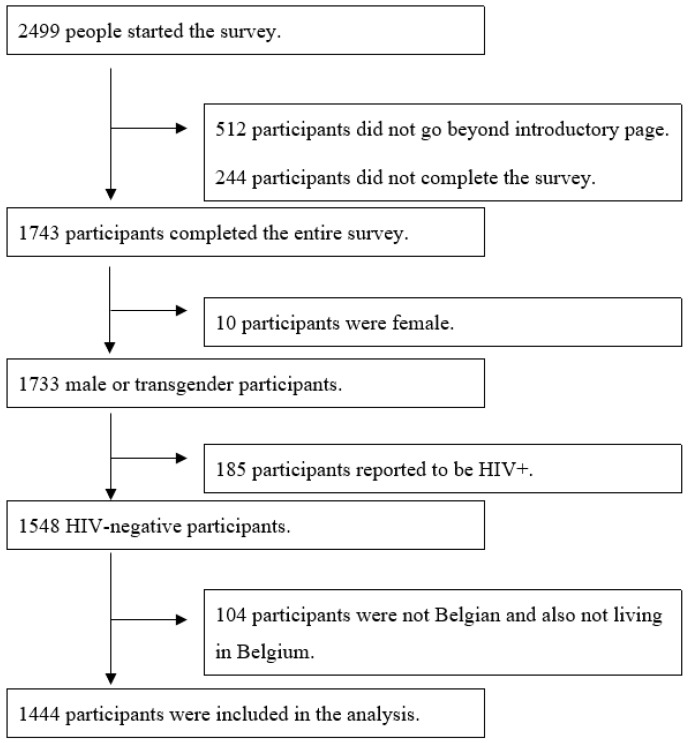
Inclusion criteria for data analysis.

We received 1444 completed questionnaires (Figure 1). Participants’ socio-demographic background characteristics are displayed in Table 1.

Participants’ median age was 36.5 years, with a minimum of 16 years and a maximum of 77 years. Almost all participants were male, except for four female-to-male transgender participants and for one person’s gender was missing. Participants were predominantly Belgian (81.2%), living in Belgium (98.1%) and highly educated (79.3%). Almost half of the participants lived in metropolitan areas, i.e., 29.8% in the Brussels capital region, and 14.4% in the Antwerp region. 

In total, 44.3% of the participants were eligible for PrEP (see Table 2). The criteria most often applied were reporting CAI with at least two different partners (33.5%), and having used psychoactive substances while engaging in sexual activities (25.3%).

Most participants (69.5%) were willing to use PrEP in the future: 84.0% of the eligible ones, and 58.0% of the illegible who were not eligible. These results will be discussed in more detail below when looking at the (in)congruence between the sub-groups.

### 3.2. Sexual and Preventive Behavior 

Most participants were sexually attracted to men (99.4%), 76 participants (5.3%) were also attracted to women (not shown in table). In the last 12 months, the median numbers of men with whom they had sex were seven, with whom they had anal intercourse five, and with whom they had CAI was one (not shown in table). About 39.8% reported that they had not engaged in any CAI with sexual partners during the last year. Sex under the influence of psychoactive drugs in the last six months was reported by 25.3%. Almost sixty percent reported having had their latest HIV test in the previous six months. PEP was used by 8.2% in the last year, 7.5% had used PrEP before. About one fifth (19.8%) perceived themselves at a high risk of acquiring an HIV infection, and 44.2% were in a steady relationship at the time of the survey.

### 3.3. PrEP Awareness, Knowledge, and Attitudes 

A great majority of the participants (91.8%) reported having been aware about PrEP (see Table 5). About 55.2% of the participants rated their PrEP knowledge as good or very good. Participants’ attitudes towards PrEP were generally positive: A vast majority perceived PrEP as a good extra prevention tool (84.9%) and agreed with the statement that “it’s a good thing that HIV negative people can protect themselves with PrEP” (90.1%). Only 15.6% felt that PrEP was unnecessary due to better alternatives. About one third of participants (33.2%) expected that PrEP users will receive negative remarks from others.

### 3.4. PrEP Eligibility and Willingness

Participants who were eligible for PrEP were significantly more likely to be willing to take PrEP (*p* < 0.001). Among those who were eligible, 16.0% were unwilling or unsure to use PrEP in the future. Among participants where were not eligible, 58% were willing to take PrEP. (Table 3). Overall, willingness was significantly associated with higher PrEP awareness (*p* < 0.001), better PrEP knowledge (*p* < 0.001), more risky sexual behavior (i.e., CAI) (*p* < 0.001), and the relationship status ‘single’ (*p* < 0.001) (results not shown in table). No significant differences were found between the four groups in terms of other socio-demographic background characteristics.

### 3.5. Eligible Participants: Factors Associated with Their Willingness to Take PrEP

Participants who were eligible for PrEP but not willing (or unsure) to use it, were significantly more likely to be in a steady relationship, to not have tested for HIV in the last six months and to have had fewer male partners for anal sex in the last 12 months when compared with eligible and willing participants: e.g., 8.8% of eligible unwilling participants reported to have had CAI with more than five partners; compared with 28.9% among eligible willing participants. Eligible unwilling participants were also less likely to perceive themselves at high risk for HIV (see Table 4). In terms of awareness-related, knowledge-related and attitudinal factors the following differences were found (see Table 5): Eligible but unwilling participants were less likely to be aware of PrEP and to consider their PrEP knowledge to be good, when compared with willing participants (*p* < 0.001). Eligible and unwilling participants were also significantly less likely to have a positive attitude towards PrEP (*p* < 0.001), although they did not significantly differ in their opinion towards the use condoms to prevent other STIs while on PrEP.

### 3.6. Ineligible Participants: Factors Associated with Their Willingness to Take PrEP

Participants who were willing to use PrEP but ineligible to do so according to the Belgian criteria were more likely to be single, to have tested for HIV in the last six months, to perceive themselves at higher risk of getting an HIV infection and had a higher number of male partners for anal sex, when compared with those ineligible and unwilling (*p* < 0.001 for these three variables; see Table 4). A substantial proportion in both groups anticipated that they may have CAI in the next three months (34.4% versus 40%, respectively). Willing participants were less likely to find it important to still use condoms when being on PrEP compared with unwilling ineligible participants (*p* < 0.001). Willing MSM were also more aware of PrEP (*p* = 0.017) and were more likely to indicate that their PrEP knowledge was (very) good, when compared with ineligible unwilling participants (*p* = 0.008; see Table 5).

## 4. Discussion

In this online survey among Belgian MSM we aimed to explore the hypothetical willingness to take PrEP and to assess it against the formal PrEP eligibility criteria. About 44.3% of the participants were eligible for PrEP according to the Belgian eligibility criteria. More than two third (69.5%) were willing to start PrEP once it became available in Belgium. More than half of those were also eligible. We also found that a small proportion of those eligible were unwilling to take PrEP (16.0%), and that more than half of those ineligible at the time of the survey were willing to take PrEP in the future (58.0%). Among eligible participants, those unwilling to take PrEP reported relative lower levels of CAI and were more often in a steady relationship. This may have contributed to their lower individual risk perception. They also had lower awareness and knowledge of PrEP, and reported less positive attitudes towards this prevention method than their willing counterparts. Among ineligible participants, a distinct group of MSM willing to take PrEP emerged, in spite of not formally qualifying for PrEP prescription: Single men perceiving themselves at risk for HIV acquisition with relative high number of male partners for anal sex. While their attitudes were more favorable towards PrEP, they were less likely to anticipate PrEP related stigma than their unwilling counterparts and anticipated to have CAI in the future. 

The study provides new information regarding PrEP uptake within a framework of formal eligibility criteria, which may be useful for other Western (European) countries facing similar situations. Our findings point to a high congruence between PrEP eligibility and hypothetical willingness to use PrEP. Most eligible MSM in our study were also willing to take PrEP, which is in line with demonstration studies showing that those coming forward for PrEP are highly likely to be at risk for HIV [20,21,23,24,25,39]. However, about 40% of participants showed incongruence between formal risk-criteria and their hypothetical willingness. A recent Australian prospective cohort study showed that 69.8% of gay and bisexual men who met the eligibility criteria had not yet commenced PrEP [40]. This is higher compared to our study where only 16% of those eligible were unwilling. On the contrary, more than half of ineligible participants were also hypothetically willing to use PrEP in the future. 

The two incongruent groups are important for HIV prevention and sexual health promotion since they require different approaches in sexual health promotion. The first group, albeit small in numbers, are the ones eligible yet unwilling to take it. This group may be concerning, given that eligibility criteria are based on factors known to be associated with high risk for HIV acquisition. The high proportion of participants in this group who had sex while using psychoactive drugs in the six months prior to the survey (52.9%) and the high proportion of participants having had CAI with at least two male partners in the last 12 months (57.8%) demonstrates that they are indeed at risk for HIV. Our data suggest that they might be unwilling (despite being eligible) to take PrEP due to an inadequate risk perception, and a less positive attitude towards PrEP. Only 10% of this group perceived themselves to be at high risk for HIV acquisition. This is in line with the lower proportion of participants that have recently tested for HIV in this group, when compared with willing, eligible participants. Overall, this may reflect misconceptions about the levels of risk required to advise PrEP use [40]. Eligible and unwilling MSM were also more likely to indicate that PrEP users will receive negative comments, potentially indicating an anticipated stigmatization of PrEP. Such anticipated social discrediting of PrEP may function as barrier in accessing PrEP [41,42,43,44,45]. 

The results suggest that this group and their sex-partners are at substantial risk for HIV acquisition, but are less likely to self-identify as being at risk and are less interested in measures such as PrEP. Future interventions should take into account that eligible, unwilling MSM are harder to reach, because they may be less likely to come forward themselves for HIV testing, counselling, and other sexual health promotion services, resulting in less opportunities for PrEP promotion [46,47,48]. Modifying HIV risk perceptions through educational interventions could be a promising strategy to promote PrEP among those who could benefit from it [49]. Also, when improving knowledge about PrEP, addressing potential negative associations is warranted. Factors related to attitudinal constructs are potentially modifiable, as are stigmatizing attitudes, and this should be considered in future interventions.

The second incongruent group concerns MSM ineligible for PrEP who were willing to take it. As the reported willingness is hypothetical, this finding does not necessarily mean that this group will come forward for PrEP despite being ineligible. However, these results do suggest that we have to be aware that there will be potential PrEP use outside of the narrow margins of clinical criteria. In Australia, it was found that consistent condom use had dropped on a community level, to a similar extent that PrEP was taken up [50]. The group we identified as ineligible but willing to take PrEP may be most prone for contributing to such a community-level risk compensation effect. However, a decreasing trend of condom use was already existing prior to the introduction of PrEP [51,52], with parallel increases of STIs [12]. PrEP is recommended for those at highest risk of HIV infection [53], but little is known about the extent of MSM starting with PrEP with the intention of using less condoms or engaging in other ‘high risk’ sexual activities. In our sample, a majority (84.1%) was convinced that it is important to keep using condoms while taking PrEP, which is a promising finding. However, we suggest further research is needed to explore to what extent PrEP remains to be perceived as an ‘additional tool’ or becoming a ‘condom substitute’ within the MSM community, to develop information strategies accordingly.

Ineligible and willing participants were more likely to perceive themselves at higher risk for HIV and to have tested for HIV in the last six months, when compared with those unwilling. Hence, it is not surprising that they were also more likely to be aware of PrEP and to self-rate their PrEP knowledge as good. The question remains whether this ineligible group is willing to take PrEP in the future should they be in need (i.e., when their HIV risk increases), or whether they are actually would be willing to take PrEP despite being at low risk, to feel better protected against HIV. Given their recent testing, such contact with the health care system provides an opportunity for counseling and helping in deciding whether or not PrEP use would be appropriate.

In contrast to our findings, it was recently observed that MSM in England self-evaluated their risk of acquiring HIV appropriately, which lead the authors to recommend PrEP for everyone perceiving themselves at risk, resulting in broader eligibility criteria [54]. The high contextuality and fluctuation of sexual (risk) behavior over time [55] justifies positioning PrEP as a positive sexual health promotion and wellness framing tool potentially avoiding PrEP-related stigma [56]. In addition, such branding and roll-out would avoid the ethical dilemma arising when denying someone PrEP coming forward for this prevention tool because of currently insufficient ‘risk behavior’ [57]. The question should perhaps not be on what comes first, reduced condom use and hence being or becoming eligible, or the intention to use PrEP. It would be unwise to deny such efficacious HIV prevention tool. Instead, we argue that the challenge lies in promoting condom use concomitantly within a combination prevention approach. 

Based on our data, we suggest that efforts need to be strengthened to promote PrEP as prevention method for MSM who are eligible for PrEP. Simultaneously, measures to endorse condoms for the prevention of STIs among PrEP users as well as a general means for effective sexual health promotion among all MSM, as stipulated by existing guidelines [58,59], is equally important.

### Limitations

Eligibility for PrEP should not be considered a static condition, because sexuality may quickly change over time in accordance with individual behavior, risky contexts, and situations leading to ‘seasons of risk” [60]. The concept of hypothetical willingness should be understood in this perspective. However, this study is the first to examine the proportion of MSM being eligible for PrEP and their willingness in an online sample in Belgium. We were able to obtain a relatively large nationwide convenience sample of 1444 participants. It may not be representative for MSM in Belgium and we cannot make inferences to the entire MSM population. The use of sexual networking applications for recruiting participants may have led to a selection bias, i.e., participants with high levels of sexual activity, seeking sex partners on the internet, or with a particular interest in PrEP. We kept the questionnaire intentionally short, so that a maximum number of participants would complete the questionnaire, although this did not allow us to obtain in-depth insights on topics such as their intentions regarding future sexual behavior and condom use. Given the likelihood of multicollinearity of several independent variables, the findings pertain to a set of covariates that are jointly related to the outcome of hypothetical willingness to use PrEP. 

## 5. Conclusions

In spite of the above limitations, we conclude that most MSM in this study were hypothetically willing to take PrEP in the future, in particular those who were eligible according to formal PrEP criteria. 

## 6. Recommendations

To increase PrEP uptake among those eligible but unwilling to take it, we recommend strategies that modify HIV risk and address potential misconceptions about PrEP. We also recommend further research to explore to what extent condom use is being replaced by PrEP on a community level, and how condoms can be promoted alongside PrEP. For PrEP to work optimally on a population level, its promotion should be embedded in a comprehensive combination prevention strategy, tailoring information and prevention needs, and including de-stigmatization of PrEP at the community level. 

## Figures and Tables

**Table 1:** Socio-demographic characteristics of participants ^a.^

Sociodemographic Characteristic	n (%)
Age	
16–25 years	177 (12.3%)
26–35 years	509 (35.2%)
36–45 years	434 (30.1%)
46+ years	324 (22.4%)
Belgian nationality (incl. dual citizenship)	1173 (81.2%)
Living in Belgium	1416 (98.1%)
Living in Brussels capital region ^b^	422 (29.8%)
Living in Antwerp metropolitan area ^b^	204 (14.4%)
Language used in interview	
Dutch	912 (63.2%)
French	359 (24.9%)
English	173 (12.0%)
Education higher than secondary school ^c^	1160 (80.5%)

^a^: *N* = 1444, ^b^: 28 missing were excluded from the analysis, ^c^: Three missing were excluded from the analysis.

**Table 2:** Eligibility criteria for PrEP in Belgium.

Eligibility Criteria	n (%)
Condomless anal intercourse with at least two different men in last six months	484 (33.5%)
More than one STI in last 12 months	75 (5.2%)
Usage of psychoactive substances while engaging in sexual activities	365 (25.3%)
At least two PEP-treatments in last twelve months	10 (0.7%)
Total number of eligible participants	639 (44.3%)

**Table 3:** Eligibility and willingness ^a.^

Willingness/Eligibility	Eligible (*n* = 639; 100%)	Not Eligible (*n* = 805; 100%)
Willing (*n* = 1004)	537 (84.0%)	467 (58.0%)
Not willing/don’t know (*n* = 440)	102 (16.0%)	338 (42.0%)

^a^ N = 1444; Chi^2^ test for association between ‘eligibiltiy’ and ‘wilingness =113.88; *p* < 0.001.

**Table 4:** Preventive and sexual behavior of participants.

Preventive and Sexual Behavior Characteristic	Total n (%) *N* = 1444	Eligible n (%)		*p*-Value ^c^	Not Eligible n (%)		*p*-Value ^c^
		Not Willing *N* = 102	Willing *N* = 537		Not Willing *N* = 338	Willing *N* = 467	
Latest HIV Test within last 6 months ^a^	869 (60.2%)	58 (56.9%)	394 (73.5%)	0.001	144 (42.6%)	273 (58.5%)	<0.001
At least one PEP usage in last 12 months	119 (8.2%)	8 (7.8%)	66 (12.3%)	0.198	15 (4.4%)	30 (6.4%)	0.226
Ever used PrEP	108 (7.5%)	5 (4.9%)	84 (15.6%)	0.004	6 (1.8%)	13 (2.8%)	0.352
Sex with psychoactive drugs in last 6 months	365 (25.3%)	54 (52.9%)	311 (57.9%)	0.352	-	-	-
Number of partners for anal sex in the last 6 months				<0.001			<0.001
0 male partners	114 (7.9%)	2 (2.0%)	5 (0.9%)		53 (15.7%)	54 (11.6%)	
1 male partner	245 (17.0%)	9 (8.8%)	9 (1.7%)		124 (36.7%)	103 (22.1%)	
2–5 male partners	479 (33.2%)	40 (39.2%)	143 (26.6%)		101 (29.9%)	195 (41.8%)	
More than 5 male partners	606 (42.0%)	51 (50.0%)	380 (70.8%)		60 (17.8%)	115 (24.6%)	
Number of partners for CAI in the last 6 months				<0.001			0.073
0 male partners	575 (39.8%)	21 (20.6%)	64 (11.9%)		218 (64.5%)	272 (58.2%)	
1 male partner	665 (46.1%)	22 (21.6%)	48 (8.9%)		120 (35.5%)	195 (41.8%)	
2–5 male partners	77 (5.3%)	50 (49.0%)	270 (50.3%)		-	-	
More than 5 male partners	127 (8.8%)	9 (8.8%)	155 (28.9%)		-	-	
Anticipate to have CAI in the next 3 months ^b^	786 (54.4%)	70 (69.3%)	413 (77.2%)	0.089	116 (34.3%)	187 (40.0%)	0.098
High self-perceived risk for HIV	286 (19.8%)	10 (9.8%)	202 (37.6%)	<0.001	15 (4.4%)	59 (12.6%)	<0.001
Currently in steady relationship	638 (44.2%)	48 (47.1%)	193 (35.9%)	0.034	192 (56.8%)	205 (43.9%)	<0.001

^a^: One missing was excluded from the analysis, ^b^: Three missing were excluded from the analysis, and ^c^: *p*-value for Chi^2^ or Fisher Exact Test for association between ‘willingness’ and characteristic; within ‘eligible’ or ‘ineligible’ groups.

**Table 5:** Awareness, knowledge and attitudes.

PrEP Awareness, Knowledge, Attitudes	Total n (%) *N* = 1444	Eligible n (%)		*p*-Value ^c^	Not Eligible n (%)		*p*-Value ^c^
		Not Willing *N* = 102	Willing *N* = 537		Not Willing *N* = 338	Willing *N* = 467	
*Awareness, knowledge*							
Heard of PrEP before (awareness)	1.326 (91.8%)	92 (90.2%)	512 (95.3%)	0.036	293 (86.7%)	429 (91.9%)	0.017
Good knowledge about PrEP ^a^	797 (55.2%)	46 (45.1%)	364 (67.8%)	<0.001	144 (42.6%)	243 (52.0%)	0.008
*Attitudes (participants partly or fully agreeing)*							
PrEP is a good extra prevention tool for HIV negative people ^b^	1.226 (84.9%)	67 (65.7%)	514 (95.7%)	<0.001	212 (62.7%)	433 (92.7%)	<0.001
PrEP is unnecessary, because there are better alternatives ^b^	225 (15.6%)	28 (27.5%)	35 (6.5%)	<0.001	115 (34.0%)	47 (10.1%)	<0.001
It’s important that PrEP users also keep using condoms to prevent other STIs ^b^	1.215 (84.1%)	82 (80.4%)	394 (73.4%)	0.136	327 (96.7%)	412 (88.2%)	<0.001
It’s a good thing that HIV negative people can protect themselves with PrEP ^b^	1.301 (90.1%)	70 (68.6%)	524 (97.6%)	<0.001	255 (75.4%)	452 (96.8%)	<0.001
I expect that PrEP users will receive negative comments ^b^	480 (33.2%)	47 (46.1%)	145 (27.0%)	<0.001	151 (44.7%)	137 (29.3%)	<0.001

^a^: Self-estimated knowledge about PrEP: ‘very good (+2)’ and ‘good (+1)’ versus ‘bad (-1)’ and ‘very bad (-2)’; ^b^: ‘totally agree (+2)’ and ‘agree (+1)’ versus ‘don’ know or unsure (0)’, ‘disagree (-1)’ and ‘totally disagree (-2)’; and ^c^: *p*-value for Chi^2^ or Fisher Exact Test for association between ‘willingness’ and characteristic. Within ‘eligible’ or ‘ineligible’ groups.
